# Protective Effects of Lithium Borate on Acute Lung Injury Induced by Intestinal Ischemia/Reperfusion in Rats

**DOI:** 10.1021/acsomega.5c11758

**Published:** 2026-04-14

**Authors:** Şule Melek, Turan Yaman, H. Turan Akkoyun, Mahire Bayramoğlu Akkoyun, Fatma Karagözoğlu, Aydın Şükrü Bengü, Ömer Faruk Keleş, Suat Ekin, Ahmet Bakır

**Affiliations:** † Bingol University, Faculty of Veterinary Medicine, Department of Surgery, Bingöl 12000, Türkiye; ‡ 53000 Van Yuzuncu Yil University, Faculty of Veterinary Medicine, Department of Pathology, Van 65090, Türkiye; § 187476 Siirt University, Faculty of Veterinary Medicine, Department of Physiology, Siirt 56100, Türkiye; ∥ Siirt University, Faculty of Veterinary Medicine, Department of Biochemistry, Siirt 56100, Türkiye; ⊥ Dokuz Eylül University, Faculty of Veterinary Medicine, Department of Zootechnique and Animal Nutrition, Bingol 12000, Türkiye; # Bingol University, Bingol Vocational School of Health Services, Bingöl 12000, Türkiye; ∇ 53000 Van Yuzuncu Yil University Department of Chemistry, Faculty of Sciences, Van 65080, Türkiye

## Abstract

The present study investigated the protective effects of lithium borate (LTB) against lung injury secondary to intestinal ischemia/reperfusion (I/R) in rats. Twenty-four male Wistar rats were allocated to four groups: control, I/R, LTB, and I/R+LTB. Intestinal ischemia was induced by superior mesenteric artery occlusion for 45 min, followed by 3 h of reperfusion. The LTB group received 15 mg/kg oral LTB for 5 days; the I/R+LTB group underwent I/R following the same LTB protocol. At the end of the experiment, lung tissue samples were taken from the rats and subjected to biochemical, histopathological, and immunohistochemical analyses. Biochemical results showed an increase in superoxide dismutase (SOD) activity, a reduction in catalase (CAT) and glutathione peroxidase (GSH-Px) activities, a reduction in glutathione (GSH) levels, and an increase in malondialdehyde (MDA) levels in the I/R group. LTB treatment reduced oxidative stress by bringing these parameters to values close to those of the control group. Histopathological examination revealed edema, hemorrhage, thickening of the interalveolar septa, and marked inflammatory cell infiltration in the I/R group, which were substantially attenuated in the I/R+LTB group. Immunohistochemical analyses showed increased Bcl-2 and caspase-3 expression in the I/R group, whereas LTB treatment was associated with reduced caspase-3 expression and modulation of Bcl-2 immunoreactivity. In conclusion, LTB attenuates oxidative stress, reduces histopathological lung damage, and suppresses apoptosis following intestinal I/R injury, suggesting its potential protective role against remote lung injury.

## Graphical abstract

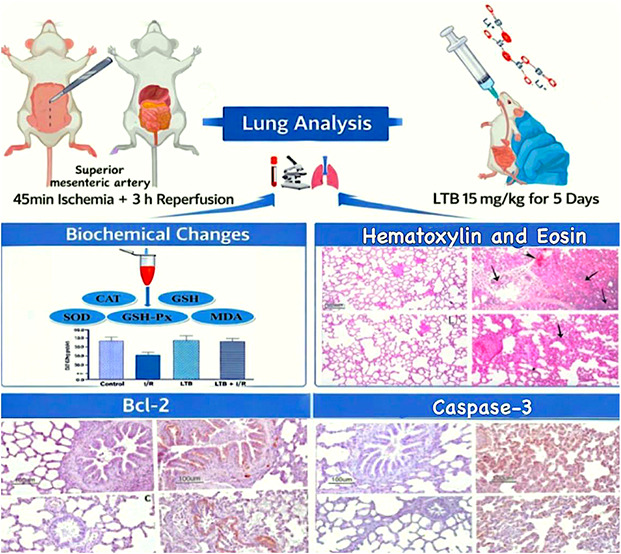

## 1 Introduction

Intestinal ischemia/reperfusion (I/R) injury is a serious condition that can occur due to certain surgical procedures, including traumatic, septic shock, serious burns, hemorrhagic, abdominal aortic surgery, small bowel transplantation, and cardiopulmonary bypass.^1^ I/R results in the deficiency of essential nutrients, especially oxygen, and activates various vascular and inflammatory mediators that promote leukocyte adhesion, migration, and activation, increase vascular permeability, and lead to a pronounced local and systemic inflammatory response and multiple organ dysfunction.^2^ I/R damage can cause indirect lung damage and contribute to the progression of acute respiratory distress syndrome.^3^ Numerous inflammatory mediators, including neutrophils, tumor necrosis factor-alpha (TNF-α), reactive oxygen species (ROS), and chemokines, have been implicated in the pathophysiology of intestinal I/R- induced tissue injury.^4^ It is well known that neutrophils that attach to injured tissues generate high amounts of ROS and thus damage the tissue.^5,6^ Clinical and experimental studies have demonstrated that oxidative stress, driven by ROS, plays a critical role as a key mediator in this process.^7^ ROS, which are generated as natural byproducts of normal cellular metabolism, have been implicated in the pathogenesis of numerous conditions, including diabetes mellitus, rheumatoid arthritis, atherosclerosis, cancer, infectious diseases, and aging.^8^ Boron is a naturally occurring element that is primarily found in the form of borate compounds within oceans, sedimentary rocks, coal deposits, and certain soils. It does not occur in its elemental state in nature, but rather exists in mineral forms such as borax, boric acid, colemanite, kernite, ulexite, and various borates.^9–12^ Borates, which are made up of boron and oxygen elements, are present in some foods at high concentrations. Borate compounds, including lithium borate, have been shown to activate endogenous antioxidant pathways, thereby mitigating oxidative stress induced by metal toxicity in both rat models and cultured cell lines.^13,14^ These compounds display antioxidant activity.^14^ Due to their antiseptic and antiepileptic properties, boron and its derivatives are employed in the development of pharmaceutical agents.^15,16^ Besides, boron compounds are commonly used in industrial, agricultural, and cosmetic applications in addition to their conventional use in healthcare services.^17^ Boron compounds are reported to have positive effects on growth^18^ bone development,^19^ hormones,^20^ brain,^21^ and cancer.^22,23^ Based on the reported antioxidant properties of LTB, the present study aimed to evaluate lung injury following intestinal I/R and to investigate whether LTB administration is associated with changes in oxidative stress parameters and histopathological findings in lung tissue.

## 2 Materials and Methods

### 2.1 Ethical Approval and Animals

Twenty-four Wistar albino male rats, weighing 200–300 g, were supplied from the experimental research center at Bingöl University, and the animal experiments started upon approval obtained from the local Ethics Committee (Meeting date: 18/05/2021; Meeting number: 2021/02; Decision number: 02/01). The rats were kept in a room at a temperature of 20 ± 1 °C with a 12-h light/dark cycle in separate cages. They were fed with food and water ad libitum. The rats were acclimatized to the environmental conditions 1 week prior to the start of the experimental procedures.

### 2.2 Experimental Design

The drug dosage and experimental method were determined in accordance with previous studies.^24,25^ A total of 24 rats were allocated randomly to 4 groups (*n* = 6).

**Control group:** The rats underwent laparotomy under anesthesia in sterile conditions without induction of intestinal I/R.

**I/R group:** The rats were positioned supine, and a midline abdominal incision was carried out. The superior mesenteric artery was carefully ligated to induce intestinal ischemia for 45 min, after which the ligature was released to allow 3 h of reperfusion.^24^

**LTB group:** 15 mg/kg (oral) LTB^25^ was given once daily for 5 consecutive days. At the end of the period, tissue samples were collected under anesthesia without induction of I/R.

**I/R+LTB group:** 15 mg/kg (oral) LTB was administered once daily for 5 consecutive days prior to intestinal I/R induction. Following treatment, the rats underwent 45 min of intestinal ischemia followed by 3 h of reperfusion, as described above. Anesthesia in the I/R and I/R+LTB groups was induced via intraperitoneal injection of xylazine hydrochloride (10 mg/kg, Rompun 2%, Bayer Turkish Chemical Industry Co. Ltd., Istanbul, Türkiye) and ketamine hydrochloride (60 mg/kg, Ketasol 10%, Richter Pharma AG, Wels, Austria). At the end of the reperfusion, a cardiac puncture was performed on all groups under anesthesia to collect blood samples. Subsequently, the rats were euthanized by decapitation in accordance with ethical rules, and lung tissues were collected.

### 2.3 Lung Tissue Preparation

Lung tissue samples weighing 0.5 g on a precision scale were placed inside tubes, and cold Tris buffer (0.32 mol/L sucrose, 10 nmol/L Tris-HCl, pH 7.4, 1 mmol/L EDTA) weighing 10 times more than 1 sample was poured into the tubes. These buffer-added samples were broken up in a homogenizer (Ultra Turrax T25, IKA, Staufen, Germany) and refrigerated at 4 °C. After vortexing, cell membrane disruption in a 20-kHz ultrasonicator was applied to the samples, which were put in porcelain crucibles, and then the samples were hydroextracted at 1600 rpm for 30 min. Clear supernatant was removed and placed into Eppendorf tubes. All of the processes were carried out at 4 °C.^26,27^

### 2.4 Antioxidant Enzyme Activity Determinations

Tissue SOD activity was determined at a wavelength of 505 nm using the procedure of Sun et al.^28^ CAT activity was assessed at a wavelength of 240 nm by the procedure explained by Aebi.^29^ GSH-Px tissue activity was evaluated at a wavelength of 340 nm by the procedure of Pagila and Valentine.^30^ Results were expressed in units of IU/mg protein. GSH was determined at a wavelength of 340 nm using DTNB using the procedure of Beutler,^31^ which is dependent on the development of a stable yellow complex. The data were expressed in mmol/mg tissue. The MDA level was examined at a wavelength of 532 nm using the procedure developed by Ohkawa et al.^32^

### 2.5 Analysis of the Total Protein Concentration

The total protein concentration in lung tissue homogenates (10–200 μg) was estimated using the method developed by Lowry et al.,^33^ with bovine serum albumin serving as the standard. Results were presented as milligrams of protein per gram of wet tissue (mg/g).

### 2.6 Histopathological Examination

Tissue samples obtained from the euthanized rats were fixed in 10% neutral buffered formalin and embedded in paraffin according to standard histological procedures. Sections of 5 μm thickness were obtained and routinely stained with hematoxylin and eosin. The stained slides were analyzed under a light microscope (E-400; Nikon Corp., Minato City, Tokyo, Japan) equipped with a DS-Ri2 video camera (DS-U3; Nikon Corp.). Lung tissues were evaluated descriptively for the presence of histopathological alterations, including edema, hemorrhage, hyperemia, airway epithelial cell damage, inflammatory cell infiltration, thickening of the interalveolar septa, and alveolar structural disruption.^34,35^ The severity of these findings was recorded using a semiquantitative grading system (negative [−], mild [+], moderate [++], severe [+++]) for comparative descriptive purposes.^6^

### 2.7 Immunohistochemical Staining

Immunohistochemical analysis was conducted according to the streptavidin–biotin–peroxidase complex procedure. Endogenous peroxidase activity was blocked by exposing the slides to 3% H_2_O_2_ (v/v) for 20 min, followed by two washes in 0.01 M of phosphate-buffered saline for 5 min each. Antigen retrieval was conducted in citrate buffer (pH 6.0) for 30 min at 95 °C in a water bath, after which the slides were allowed to cool for 20 min. Nonspecific binding was minimized by incubating the sections with blocking serum (Histostain Plus Bulk Kit; Zymed Laboratories Inc., Oxnard, CA, USA) for 15 min. The tissue sections were then incubated overnight at 4 °C with primary antibodies against Bcl-2 (1:100 dilution, 59348; Abcam, Waltham, MA, USA) and caspase-3 (1/100 dilution, PA5–16335; Thermo Fisher Scientific Inc., Waltham, MA, USA). Subsequently, the slides were incubated with a biotinylated secondary antibody (Histostain Plus Bulk Kit; Zymed Laboratories Inc.) for 20 min at room temperature, followed by incubation with streptavidin-peroxidase (HRP) conjugate (Histostain Plus Bulk Kit; Zymed) for an additional 20 min at room temperature. Visualization was achieved using diaminobenzidine for 5–15 min. The slides were then rinsed with distilled water (3 × 5 min), counterstained with Gill’s hematoxylin for 3 min, dehydrated with a graded series of alcohol and xylene, and finally mounted with Entellan mounting medium. Immunohistochemical staining was evaluated descriptively based on the relative intensity of staining in the tissue sections (negative [−], mild [+], moderate [++], severe [+++]) under light microscopy with a DS-Ri2 digital camera. This evaluation reflects overall immunoreactivity rather than quantitative protein expression levels.

## 3 Statistical Analyses

Data are given as means ± the standard error of the mean (x ± SEM). Statistical analyses were performed using either the Kruskal–Wallis test or one-way analysis of variance (ANOVA), as appropriate. Posthoc comparisons of group means were conducted with Tukey’s test. A *p* value less than 0.05 was considered statistically significant. All statistical analyses were conducted with IBM SPSS Statistics for Windows 23.0 (IBM Corp., Armonk, NY, USA). Sample size was determined by an a priori power analysis using G*Power software (version 3.1), with a conservative large effect size (*f* = 0.90), in accordance with accepted methodological recommendations for experimental animal studies.

## 4 Results

### 4.1 Biochemical Findings

Intestinal I/R significantly increased SOD activity (Figure 1) and MDA levels (Figure 5) while decreasing CAT (Figure 2) and GSH-Px activities (Figure 3) and GSH levels (Figure 4) compared to the control group. LTB treatment restored antioxidant enzyme activities and reduced lipid peroxidation, bringing these parameters closer to control values (Figure 5).

**Figure 1:**
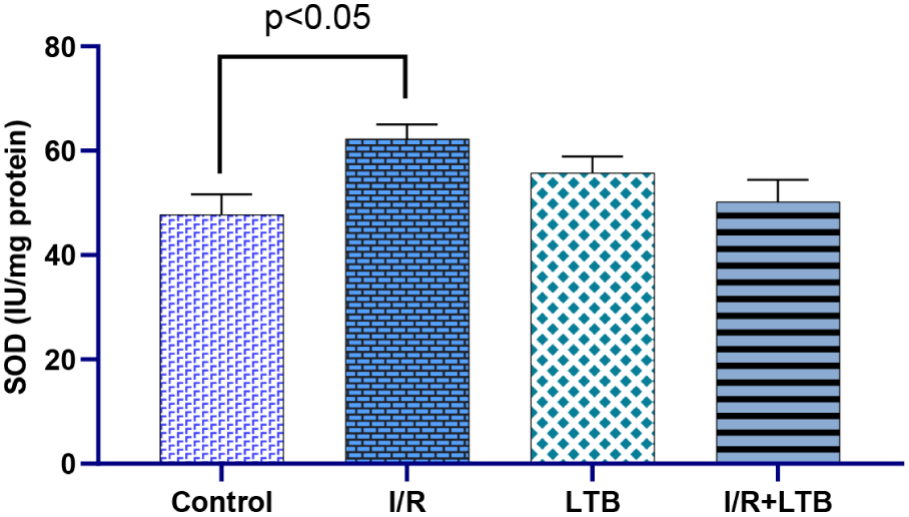
SOD enzyme activity in lung tissue samples of the control, I/R, LTB, and I/R+LTB groups. Effect sizes with 95% CIs analysis (Cohen’s d) of control vs I/R, I/R vs I/R+LTB, and control vs I/R+LTB groups revealed that SOD (effect size = −1.818, 95% CI: −3.146 to −0.439; effect size = 0.608, 95% CI: −0.296 to 1.464; and effect size = −0.469, 95% CI: −1.298 to 0.400). *n* = 6 per group.

**Figure 2:**
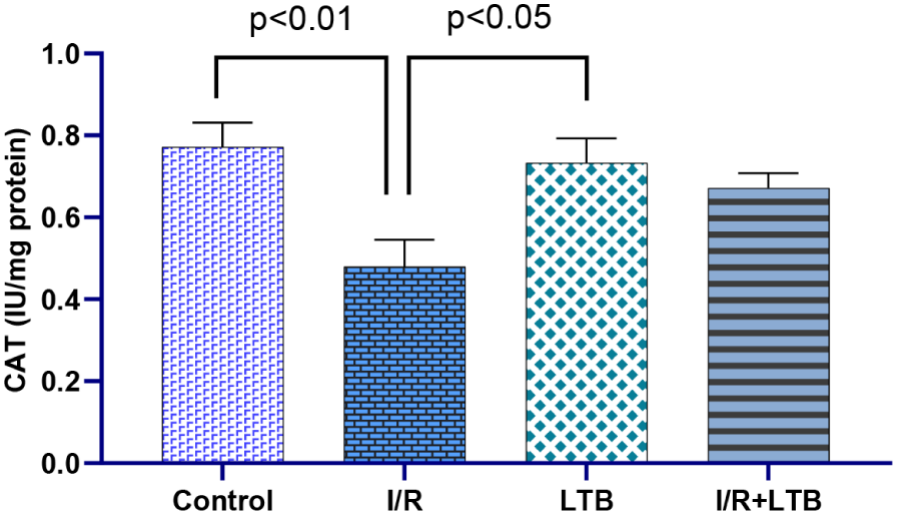
CAT enzyme activity in lung tissue samples of the control, I/R, LTB, and I/R+LTB groups. Effect sizes with 95% CIs analysis (Cohen’s d) of control vs I/R, I/R vs I/R+LTB, and control vs I/R+LTB groups showed that CAT (effect size = 1.105, 95% CI: 0.0343 to 2.1155; effect size = −1.210, 95% CI: −2.2619 to −0.098; and effect size = 0.542, 95% CI: −0.3442 to 1.3846). *n* = 6 per group.

**Figure 3:**
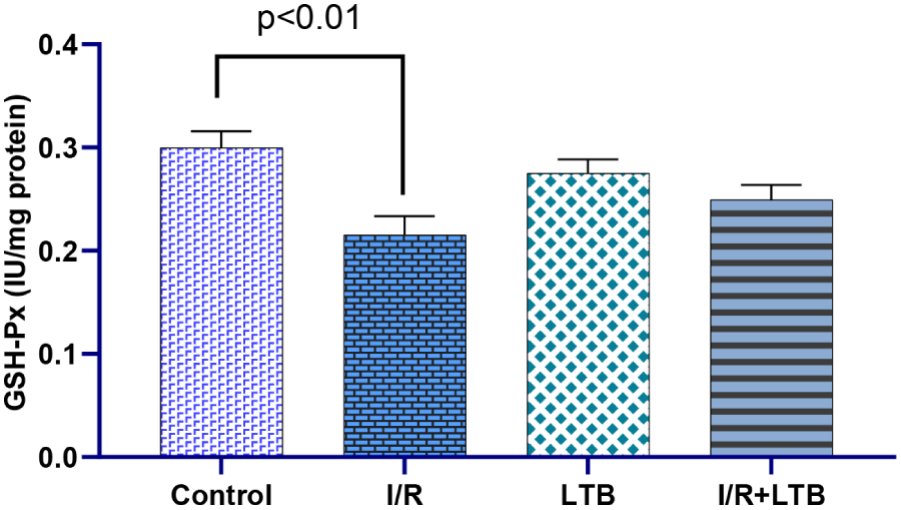
GSH-Px enzyme activity in lung tissue samples of the control, I/R, LTB, and I/R+LTB groups. Effect sizes with 95% CIs analysis (Cohen’s d) of control vs I/R, I/R vs I/R+LTB, and control vs I/R+LTB groups found that GSH-Px (effect size = 2.471, 95% CI: 0.769 to 4.1359; effect size = −0.913, 95% CI: −1.855 to 0.0871; and effect size = 0.810, 95% CI: −0.155 to 1.7195). *n* = 6 per group.

**Figure 4:**
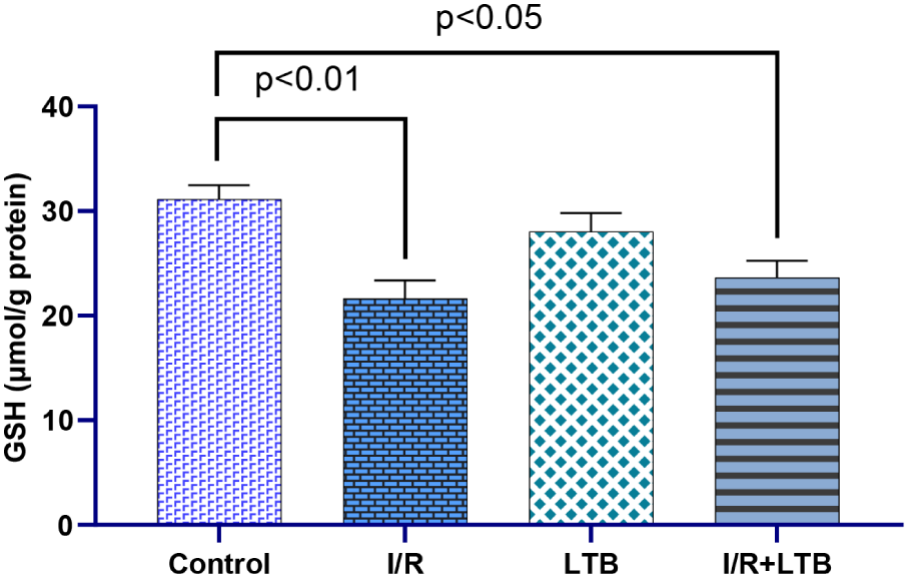
GSH level in lung tissue samples of the control, I/R, LTB, and I/R +LTB groups. Effect sizes with 95% CIs analysis (Cohen’s d) of control vs I/R, I/R vs I/R+LTB, and control vs I/R+LTB groups indicated that GSH (effect size = 1.852, 95% CI: 0.456 to 3.196; effect size = −0.879, 95% CI: −1.810 to 0.109; and effect size = 1.463, 95% CI: 0.245 to 2.624). *n* = 6 per group.

**Figure 5:**
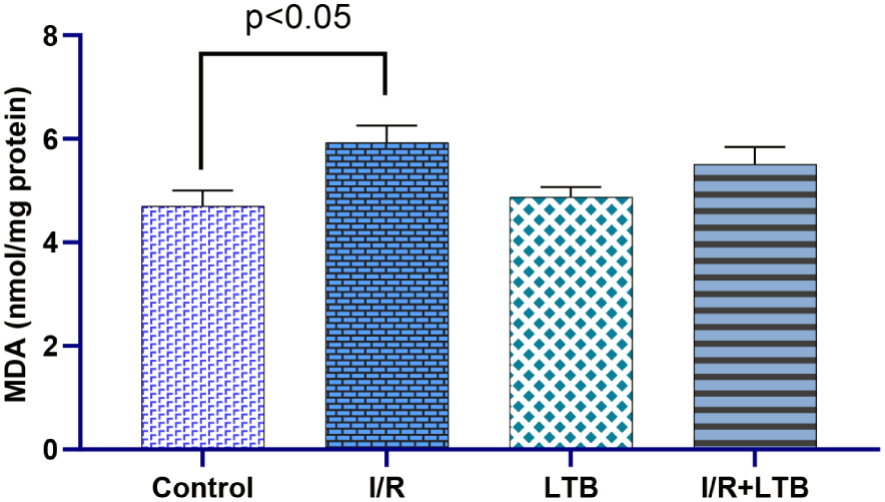
MDA levels in lung tissue samples of the control, I/R, LTB, and I/R+LTB groups. Effect sizes with 95% CIs analysis (Cohen’s d) of control vs I/R, I/R vs I/R+LTB, and control vs I/R+LTB groups demonstrated that MDA (Effect size = −0.955, 95% CI: −1.91062 to 0.0601; effect size = 1.037, 95% CI: −0.00787 to 2.020; and effect size = −0.722, 95% CI: −1.60580 to 0.2156). *n* = 6 per group.

### 4.2 Histopathological Findings

Examination of lung tissue samples from the control group revealed a normal histological architecture (Figure 6a). In contrast, rats in the I/R group presented hemorrhage, edema, and infiltration of inflammatory cells, predominantly neutrophils. Thickening of the interalveolar septum was also observed. In certain areas, large and irregular air spaces were identified due to the degradation of interalveolar septa (Figure 6b) The LTB group exhibited histological features generally comparable to those observed in the control group (Figure 6c). In the I/R+LTB group, these histopathological alterations appeared less pronounced in comparison to the untreated I/R group (Figure 6d). A semiquantitative evaluation of histopathological lung injury scores, presented as median (interquartile range), along with statistical comparisons among groups, is summarized in Table 1.

**Table 1:** Incidence and Severity of Histopathological Lung Lesions among Control, I/R, LTB, and I/R+LTB Groups^a^

Changes/lesions in lung	Control	I/R	LTB	I/R+LTB	*p*
Edema	0 (0–0)^a^	2 (1–2)^b^	0 (0–0)^a^	0 (0–1)^a^	<0.001
Hemorrhage	0 (0–0)^a^	1.5 (1–3)^b^	0 (0–0)^a^	0.5 (0–1)^a^	0.007
Inflammatory cell infiltration	0 (0–0)^a^	2.5 (2–3)^b^	0 (0–0)^a^	0 (0–1)^a^	<0.001
Epithelial damage/disruption	0 (0–0)^a^	1.5 (1–2)^b^	0 (0–0)^a^	0 (0–1)^a^	0.005
Thickness in interalveolar septum	0 (0–0)^a^	2 (1–2)^b^	0 (0–0)^a^	0 (0–1)^a^	<0.001

a Data are presented as median (Q1–Q3). Severity scoring: 0 = negative, 1 = mild, 2 = moderate, 3 = severe. The Kruskal–Wallis test followed by Dunn’s multiple comparison test with Bonferroni correction was used. Different superscript letters (a,b) within the same row indicate statistically significant differences (*p* < 0.05).

**Figure 6:**
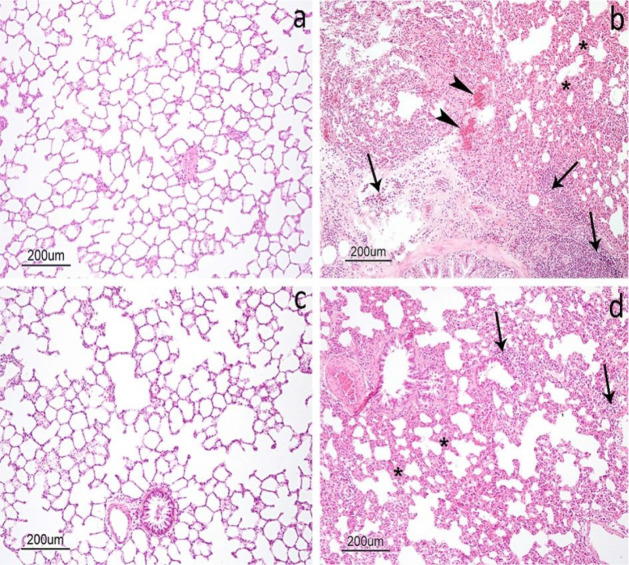
Cross-section of the lung tissue stained with hematoxylin and eosin (H&E): a) control group: normal histological appearance of the lung with normal alveoli and thin interalveolar septa; b) I/R group: inflammatory cell infiltration (arrows), hemorrhage (arrowheads), and thickening of the alveolar wall (stars) are observed; c) LTB group: normal lung architecture; and d) I/R+LTB group: less inflammatory cell infiltration (arrows) and alveolar wall thickness are observed (stars).

### 4.3 Immunohistochemical Findings

Bcl-2 immunoreactivity was not observed in the lung tissue sections of the control group (Figure 7a). In contrast, cytoplasmic Bcl-2 immunoreactivity was detected in bronchial and bronchiolar epithelial cells in the I/R group (Figure 7b). Similarly, no evident Bcl-2 immunoreactivity was observed in the LTB-only group (Figure 7c). In the I/R+LTB group, Bcl-2 immunoreactivity was present and appeared more widespread compared to the I/R group (Figure 7d). Consistent with these observations, semiquantitative analysis revealed significant differences in Bcl-2 immunoreactivity among the experimental groups (*p* < 0.001) (Table 2). While no Bcl-2 expression was detected in the control and LTB groups, the I/R group demonstrated moderate cytoplasmic expression in bronchial and bronchiolar epithelial cells. Notably, the I/R+LTB group exhibited stronger and more extensive Bcl-2 immunoreactivity compared with the I/R group.

**Figure 7:**
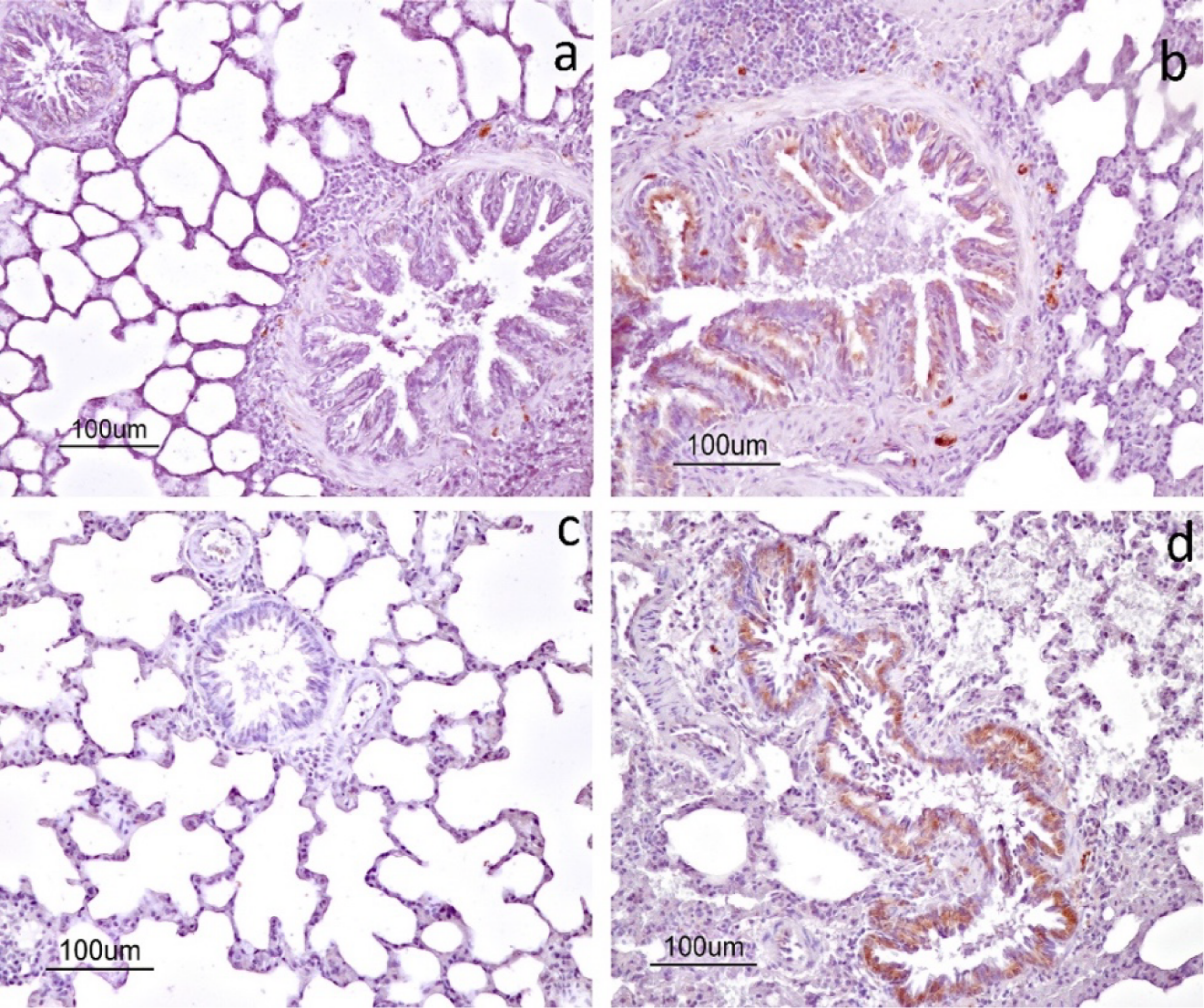
Immunoexpression of Bcl-2 within the lung tissue of the rats: a) control group; b) I/R group; c) LTB group; and d) I/R+LTB group. ABC method, counterstained with hematoxylin.

**Table 2:** Semi-Quantitative Evaluation of Bcl-2 Immunoreactivity in Lung Tissue^a^

Group	Negative (0)	Mild (1)	Moderate (2)	Severe (3)	Median (Q1–Q3)
Control	6	0	0	0	0 (0–0)
I/R	0	1	3	2	2 (2–3)
LTB	6	0	0	0	0 (0–0)
I/R+LTB	0	0	2	4	3 (2–3)

a Immunoreactivity was semiquantitatively scored as follows: 0 = negative, 1 = mild, 2 = moderate, 3 = severe staining. Data are presented as median (Q1–Q3).

Caspase-3 immunoreactivity was not observed in the lung tissue sections of the control group (Figure 8a). In contrast, strong caspase-3 immunoreactivity was detected in various lung tissue cells in the I/R group (Figure 8b). Similarly, no evident caspase-3 immunoreactivity was observed in the LTB group (Figure 8c). In the I/R+LTB group, caspase-3 immunoreactivity was present but appeared less extensive compared with that of the I/R group (Figure 8d). Consistent with these findings, semiquantitative analysis demonstrated significant differences in caspase-3 immunoreactivity among the experimental groups (*p* < 0.001) (Table 3). While no caspase-3 expression was detected in the control and LTB groups, the I/R group showed strong immunoreactivity in lung tissue cells. In contrast, the I/R+LTB group exhibited reduced caspase-3 immunoreactivity compared to the I/R group.

**Figure 8:**
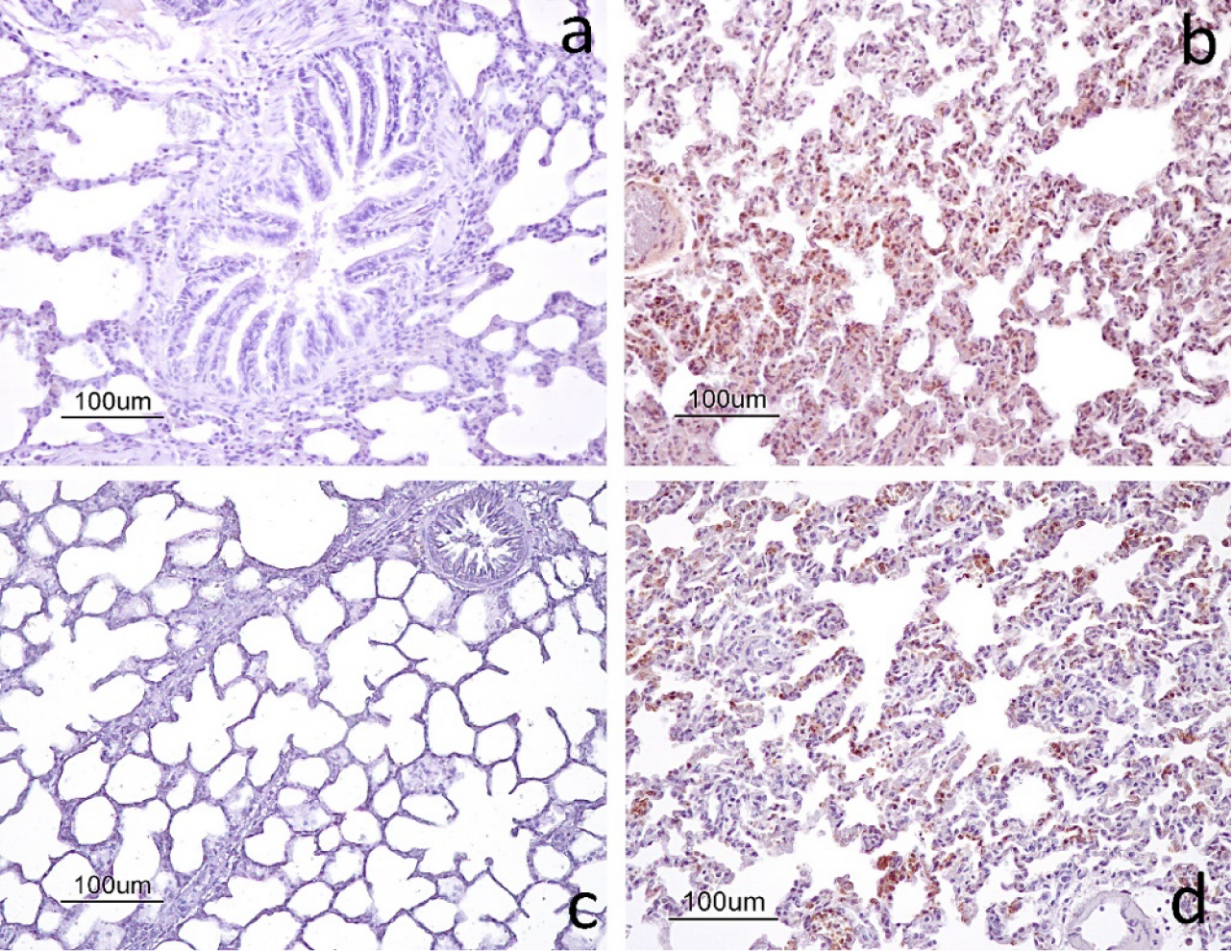
Immunoexpression of caspase-3 within the lung tissue of the rats: a) control group; b) I/R group; c) LTB group; and d) I/R+LTB group. ABC method, counterstained with hematoxylin.

**Table 3:** Semi-Quantitative Evaluation of Caspase-3 Immunoreactivity in Lung Tissue^a^

Group	Negative (0)	Mild (1)	Moderate (2)	Severe (3)	Median (Q1–Q3)
Control	6	0	0	0	0 (0–0)
I/R	0	1	2	3	3 (2–3)
LTB	6	0	0	0	0 (0–0)
I/R+LTB	1	3	2	4	1 (1–2)

a Immunoreactivity was semiquantitatively scored as follows: 0 = negative, 1 = mild, 2 = moderate, 3 = severe staining. Data are presented as median (Q1–Q3).

## 5 Discussion

Intestinal I/R is considered a critical and important event in the improvement of organ dysfunction.^5^ Although intestinal ischemia injury occurs due to lack of sufficient circulation, the real injury takes place during reperfusion period.^36^ I/R injury originates from the free radical formation with the reoxygenation of the tissue after reperfusion.^6^ I/R injury, which occurs in range of medical conditions such as intestinal ischemia and myocardial infarction, constitutes a significant pathological process and is among the primary contributors to mortality linked to chronic illness on a global scale.^36^ Mechanisms and treatments of intestinal I/R were extensively covered as subjects of other research. Even though adequate light has not been shed on its mechanism, it is thought that ROS and inflammatory pathways play significant roles in such mechanisms.^37^ ROS and apoptosis associated with I/R lead to significant damage in intestinal epithelial cells. Moreover, ROS-induced inflammatory responses, which involve the production of pro-inflammatory cytokines and oxygen-derived free radicals, can exacerbate intestinal injury.^38^ ROS also can react with proteins, nucleic acids, and lipids, causing lipid peroxidation of biological membranes, and can create lipid peroxidation such as MDA.^39,40^ Although many ROS are produced during oxidative stress, body antioxidants can protect the body’s cells and tissues against ROS attacks only so far.^41^ The antioxidant defense system counteracts ROS-induced damage through enzymatic components such as SOD, CAT, and GSH-Px, as well as nonenzymatic molecules like GSH. Accordingly, recent research has demonstrated that antioxidant administration can attenuate oxidative stress and modulate both local and systemic inflammatory responses associated with intestinal I/R injury.^42^ MDA, on the other hand, which is the last product of lipid peroxidation, occurs after the exposure of cell lipid acids to radicals.^43,44^ MDA is a commonly used oxidative stress indicator.^45,46^ The aim of this study was to investigate the effects of I/R and LTB application on significant antioxidant enzymes such as SOD, CAT, and GSH-Px, as well as GSH and MDA levels. It was seen that the SOD enzyme activity increased in the I/R group in comparison to the control group, and this increase was statistically significant (*p* < 0.05). However, the enzyme activity reduced in the LTB group in comparison to the I/R group and approached the values of the control group. When the CAT enzyme activity was evaluated, the enzyme activity decreased (*p* < 0.01) in the I/R group in comparison to the control group. This decrease is thought to be an indicator for the damage that occurred as a result of the I/R. In the I/R+LTB group, the enzyme activity was closer to that of the control group. However, no statistical difference was observed between the values. GSH-Px enzyme activity in the I/R group was found to decrease (*p* < 0.01) compared to the control group due to the injury. In the LTB group, the enzyme activity values were closer to those in the control group. Tissue SOD enzyme activity significantly increased in the I/R group in the current study. The increase in tissue SOD enzyme activity could be well related to the rise in oxidative stress as reflected by elevated tissue MDA levels leading to tissue damage. It was observed that the rise in tissue SOD enzyme activity in the I/R group was related to oxidative I/R damage and was then restored by LTB administration. GSH, GSH-Px, and CAT were depleted from lung tissue due to I/R oxidative injury these were restored by LTB administration. These findings support the protective role of LTB against I/R-induced oxidative lung injury. The GSH value in the study was observed to decrease similar to the enzyme activity decrease values of the I/R group (*p* < 0.01), and this decrease was statistically significant. The LTB application prevented the decrease in GSH values. This study also evaluated the MDA level, which is an especially significant product of lipid peroxidation. One of the most crucial consequences of the damage incurred by free oxygen radicals to the tissues is lipid peroxidation. It was determined that the MDA level increase in the I/R group was based on the injury. The rise was found to be statistically significant (*p* < 0.05). The MDA level was relatively increased in the LTB group as compared to the control group; however, this increase was much less in comparison to the I/R group. A different study suggested intestinal I/R can cause pneumonia, and compounds with antioxidant characteristics can be used to prevent this injury.^47^ Koike et al. (1995) showed that intestinal I/R triggers lung injury through polymorphonuclear neutrophil, which is characterized by an increasing microvascular leakage in the lungs.^48^ A separate study investigated the protective effects of hydrogen-rich saline against lung injury caused by intestinal I/R in rat models. Following 90 min of intestinal ischemia and 4 h of reperfusion, there was a notable increase in neutrophil infiltration, lipid membrane peroxidation of cell membranes, and activation of nuclear factor-kappaB (NF-κB) in lung tissue.^3^ Ito et al. (2005) reported that intestinal I/R plays a crucial role in the progression of distant organ dysfunction, most notably affecting the lungs.^5^ As a result, respiratory failure is a common complication and a leading cause of mortality following intestinal I/R. They stated that when subjected to 180 min of intestinal ischemia, the rats showed a very high death percentage within 24 h. Another study underscored the lungs as among the most susceptible organs affected by intestinal I/R. The application of intestinal I/R was found to increase MDA levels and pathological scores, while also promoting both intestinal and pulmonary damage, marked by elevated expressions of myeloperoxidase (MPO), TNF-α, intercellular adhesion molecule-1 (ICAM-1), and NF-κB.^4^ Another study showed that lung tissue injury incurred by intestinal I/R drastically increased with MPO activity, which is observed with pathology and BALF protein, interleukin-6 (IL-6) level, and ICAM-1 expression, and the NF-κB increase and SOD activity simultaneously decreased.^49^ It was reported that lung injury induced by intestinal I/R led to significant increases in MDA levels, MPO activity, caspase-3 expression, SOD activity, and p-Akt expression.^50^ Studies investigating the impact of lithium tetraborate, a boron-based compound with antioxidant and anti-inflammatory properties, on I/R injury are relatively scarce in the literature. However, one study demonstrated that lithium tetraborate may reduce oxidative stress-induced endothelial dysfunction and the inflammatory response in cardiac cells, thereby mitigating both apoptosis and necrosis in myocytes.^51^ Another study suggested that LTB can have positive effects on the liver and kidney based on the dosage used.^25^ Another study reported that LTB mitigated oxidative stress by regulating oxidative stress biomarkers and maintaining the oxidant/antioxidant balance via the Nrf2/HO-1 and NF-κB signaling pathways.^14^ Accumulation of neutrophils in the lungs was reported to be a distinctive characteristic of lung injury after intestinal I/R.^52^ In cases of lung injury triggered by intestinal I/R, the accumulation of neutrophil leukocytes and their enzymatic byproducts within lung tissue contributes to heightened microvascular permeability, along with perivascular and interstitial edema, ultimately leading to the development of pulmonary edema.^53,54^ The present study identified the presence of inflammatory cells in the I/R group. Other findings identified such as hemorrhage, and alveolar wall thickening complied with the previous studies done on I/R-induced acute lung injury.^55,56^ It was stated that decreasing immune response is crucial to prevent acute lung injury from occurring due to I/R.^34^ Additionally, it was revealed that certain antioxidants can display various protective effects for I/R injury.^56,57^ Recoveries in the treatment group might have originated from the anti-inflammatory and antioxidant characteristics of LTB.

Apoptosis is a key mechanism associated with intestinal ischemia–reperfusion (I/R) injury and plays a critical role in both the onset and progression of tissue damage.^58^ The Bcl-2 family of proteins, including Bax and Bcl-2, plays a central role in the regulation of the apoptotic process.^59^ Bax is a proapoptotic protein, whereas Bcl-2 functions as an antiapoptotic counterpart.

Previous experimental studies have demonstrated that intestinal I/R injury induces apoptotic signaling not only in the intestine but also in distant organs such as the lungs. In a rat model, Wang et al. (2015) reported a marked increase in Bcl-2 immunoexpression in alveolar, bronchial, and epithelial cells of lung tissue following I/R injury, while no expression was detected in the control group.^60^ The findings of the present study are consistent with these results as Bcl-2 expression was absent in the control group and increased in the I/R group.

Although Bcl-2 is an antiapoptotic protein, its increased expression in the I/R group should not be interpreted as an indicator of reduced apoptosis. Instead, this increase likely represents a compensatory cellular response to severe apoptotic stress induced by ischemia–reperfusion. I/R injury triggers strong proapoptotic signaling pathways, including mitochondrial dysfunction and oxidative stress, and the upregulation of Bcl-2 reflects an intrinsic survival attempt by injured cells, which is often insufficient to prevent apoptosis. This interpretation is supported by the concurrent marked increase in caspase-3 expression observed in the I/R group in the present study.

In the I/R+LTB group, Bcl-2 immunoreactivity appeared more widespread compared to that of the I/R group, while caspase-3 immunoreactivity was descriptively less extensive. Caspase-3 is frequently used in experimental studies as an immunohistochemical marker associated with apoptotic processes.^61,62^ In line with previous reports indicating increased caspase-3 activity during the reperfusion period,^63^ the present study demonstrated prominent caspase-3 immunoreactivity in the I/R group. The comparatively reduced staining observed in the I/R+LTB group suggests that LTB administration was associated with altered immunoreactivity patterns related to cell injury responses.

Taken together, the concurrent differences observed in Bcl-2 and caspase-3 immunostaining between the I/R and I/R+LTB groups may indicate that LTB influences the cellular response patterns in lung tissue following intestinal I/R injury. However, because molecular analyses and functional assays were not performed, these findings should be regarded as descriptive immunohistochemical observations. Further studies using quantitative and mechanistic approaches are required to clarify whether these staining patterns correspond to the modulation of apoptotic pathways.

### 5.1 Limitations

Several limitations should be acknowledged. First, direct inflammatory mediators such as TNF-α, ICAM-1, NF-κB, or MPO were not quantitatively assessed. Second, only a single LTB dose and a single reperfusion time point were evaluated, precluding dose–response and temporal analyses. Third, only male rats were used; therefore, sex-dependent effects cannot be excluded. Finally, apoptosis assessment relied on Bcl-2 and caspase-3 without additional proapoptotic markers such as Bax. Future studies that address these limitations are warranted.

## 6 Conclusion

LTB exerts protective effects against lung injury secondary to intestinal I/R by restoring antioxidant defenses, reducing lipid peroxidation, attenuating histopathological damage, and suppressing apoptosis. Although direct inflammatory mediators were not quantitatively assessed, the observed improvement in histopathological features associated with inflammation suggests an indirect protective effect. Overall, these findings indicate that LTB may represent a promising therapeutic candidate for mitigating remote organ injury following intestinal I/R.
